# Myeloid-Specific Deletion of Peptidylarginine Deiminase 4 Mitigates Atherosclerosis

**DOI:** 10.3389/fimmu.2018.01680

**Published:** 2018-07-26

**Authors:** Yudong Liu, Carmelo Carmona-Rivera, Erica Moore, Nickie L. Seto, Jason S. Knight, Milton Pryor, Zhi-Hong Yang, Saskia Hemmers, Alan T. Remaley, Kerri A. Mowen, Mariana J. Kaplan

**Affiliations:** ^1^Systemic Autoimmunity Branch, National Institute of Arthritis and Musculoskeletal and Skin Diseases (NIAMS), National Institutes of Health (NIH), Bethesda, MD, United States; ^2^Division of Rheumatology, Department of Internal Medicine, University of Michigan, Ann Arbor, MI, United States; ^3^Lipoprotein Metabolism Section, National Heart, Lung, and Blood Institute, NIH, Bethesda, MD, United States; ^4^The Scripps Research Institute, La Jolla, CA, United States

**Keywords:** atherosclerosis, neutrophil extracellular traps, peptidylarginine deiminase 4, inflammation, macrophages

## Abstract

Increasing evidence suggests that neutrophil extracellular traps (NETs) may play a role in promoting atherosclerotic plaque lesions in humans and in murine models. The exact pathways involved in NET-driven atherogenesis remain to be systematically characterized. To assess the extent to which myeloid-specific peptidylarginine deiminase 4 (PAD4) and PAD4-dependent NET formation contribute to atherosclerosis, mice with myeloid-specific deletion of PAD4 were generated and backcrossed to Apoe^−/−^ mice. The kinetics of atherosclerosis development were determined. NETs, but not macrophage extracellular traps, were present in atherosclerotic lesions as early as 3 weeks after initiating high-fat chow. The presence of NETs was associated with the development of atherosclerosis and with inflammatory responses in the aorta. Specific deletion of PAD4 in the myeloid lineage significantly reduced atherosclerosis burden in association with diminished NET formation and reduced inflammatory responses in the aorta. NETs stimulated macrophages to synthesize inflammatory mediators, including IL-1β, CCL2, CXCL1, and CXCL2. Our data support the notion that NETs promote atherosclerosis and that the use of specific PAD4 inhibitors may have therapeutic benefits in this potentially devastating condition.

## Introduction

Atherosclerosis is initiated by chronic injury to the endothelium and subsequent accumulation of a rich population of innate and adaptive immune proinflammatory cells, which secrete pathogenic mediators, amplify local inflammation, and promote thrombotic complications. Recently, neutrophils have emerged as potentially indispensable players in the pathogenesis of atherosclerosis. Neutrophils are detected in the subendothelial and intima compartments in both early and advanced plaque lesions and contribute to atherogenesis and plaque destabilization (1–3). Neutrophils are highly activated in acute coronary syndromes and undergo enhanced formation of neutrophil extracellular traps (NETs) at culprit lesion sites (4). NETs are composed of extracellular web-like decondensed chromatin bound to neutrophil-derived proteins. These structures have been identified in the arterial lumen in murine and human plaques and are implicated in the pathogenesis of atherosclerosis through pleiotropic mechanisms (1–3, 5, 6).

Neutrophil extracellular traps can be induced by a number of factors, including pathogens and sterile inflammatory stimuli such as cytokines, immune complexes, crystals, and autoantibodies (7). The enzyme peptidylarginine deiminase 4 (PAD4) (8, 9) is expressed in mouse and human neutrophils and can acquire a nuclear localization where, during NET formation, mediates histone citrullination contributing to chromatin decondensation. These events have been considered a hallmark of NET formation and PAD4 has therefore been proposed to play an indispensable role in NET formation following certain types of stimulation (8). In the C57Bl/6 background, PAD4-deficient mice are impaired in their ability to form NETs, whereas chemical inhibition of PAD4 abrogates NET formation in both human and mouse neutrophils (5, 9–11).

We and others have investigated the role of NETs in the development of vascular damage and atherosclerosis (5, 6). By inhibiting PAD activity utilizing the pan-PAD chemical inhibitor Cl-amidine, we found that this compound blocked NET formation, modulated inflammatory responses in the arterial plaque, reduced atherosclerotic lesion area and plaque burden and decreased the prothrombotic phenotype of Apolipoprotein-E knockout mice (Apoe^−/−^). These improvements were accompanied by reduced recruitment of netting neutrophils and macrophages to arteries and reduced arterial type-I interferon responses (5). However, Cl-amidine also blocks other PADs besides PAD4 and could potentially have other off-target effects or modulate other cell types beyond myeloid cells. Indeed, recent evidence has indicated that PAD-mediated hypercitrullination can affect T cell polarization and cytokine production (12). PAD inhibition can downregulate dendritic cell activation and proinflammatory cytokine production (13, 14). Different groups have questioned the relative role of PAD4, neutrophil elastase (NE), and/or other molecules in driving NETosis and whether NETs play important roles in driving atherosclerosis (15, 16). Thus, the role of myeloid-derived PAD4 in vascular damage and atherosclerosis remains to be better defined. We have now assessed the role of myeloid-specific PAD4 and PAD4-dependent NETs in the development of atherosclerosis.

## Materials and Methods

### Mice and Atherosclerosis Model

All mice were on the C57Bl/6 background. Apoe^−/−^ (B6.129P2-Apoetm1Unc/J) mice were purchased from The Jackson Laboratory (Bar Harbor, ME, USA). The generation of the PAD4^flox/flox^ mice has been previously described (17). PAD4^flox/flox^ mice were first backcrossed to Apoe^−/−^ mice to generate Apoe^−/−^PAD4^flox/flox^ mice. Apoe^−/−^PAD4^flox/flox^ mice were further crossed with transgenic mice expressing Cre recombinase under the control of the LysM promoter [LysMCre, B6.129P2-Lyz2tm1(cre)Ifo/J] to generate Apoe^−/−^LysM^−/−^ PAD4^flox/flox^ mice (WT) or Apoe^−/−^LysMCre^+/−^PAD4^flox/flox^ mice (PAD4 KO) littermates. The Apoe^−/−^PAD4^flox/flox^ mice without the Cre recombinase were used as controls throughout the study. All the mice were maintained in specific-pathogen-free conditions and used in accordance with NIH guidelines under the NIAMS-approved animal study protocol #A016-05-23. For studies of atherosclerosis, 8-week-old female WT or PAD4 KO mice were fed high-fat chow (HFC) (Envigo TD.88137, 0.2% cholesterol, 42% from fat-adjusted calorie diet, Indianapolis, IN, USA) for indicated time points, as previously described (18). For deoxyribonuclease (DNase) I treatment, 8-week-old female WT or PAD4 KO mice were fed HFC for a total of 6 weeks. From the third week of HFC, mice were intravenously injected 400 U DNase I (Sigma-Aldrich) in 200 µl PBS or 200 µl PBS alone as control, three times weekly for additional 4 weeks.

### Mice Neutrophil Isolation

Bone marrow (BM) neutrophils were isolated as previously described (5). Briefly, BM was flushed from femurs and tibias, and total cells were overlayed on a discontinuous Percoll gradient (52, 69, and 78%) at 2,000 × *g* for 30 min. Cells were collected from the 69 to 78% interface, and RBCs were lysed with ACK Lysing Buffer (Quality Biological). Cells were >95% Ly-6G-positive by flow cytometry, and had the typical nuclear morphology by microscopy (data not shown). Peritoneal exudate neutrophils were obtained by peritoneal lavage from 8- to 10-week-old WT or PAD4 KO mice 16 h after i.p. injection with sterile thioglycollate (Remel), as described (19), followed by fluorescence-activated cell sorting (FACS) based on the expression of CD45, CD11b, and Ly-6G.

### Detection of NETs by Immunofluorescence Microscopy

Fluorescence-activated cell sorting-sorted peritoneal neutrophils (1 × 10^6^ cells/ml) were pre-incubated in poly-l-lysine coated cover slips for 15 min, supernatants were removed, and indicated stimuli were added [calcium ionophore, A23187 (10 µM), EMD Millipore, Billerica, MA, USA] for 4 h at 37°C. After incubation, cover slips were fixed with 4% paraformaldehyde, and blocked with 0.2% gelatin for 30 min. Cells were stained with rabbit anti-citrullinated histone 3 (Abcam, ab 5103) for 1 h, followed by staining with Alexa Fluor 488-conjugated donkey anti-rat IgG and Alexa Fluor 555-conjugated donkey anti-rabbit IgG (Life Technologies). After mounting (Prolong, Life Technologies), cells were visualized by confocal microscopy.

### Characterization and Quantification of Atherosclerosis

Atherosclerosis was assessed as previously described (20, 21). Briefly, after euthanasia, the vasculature was perfused with PBS. For aortic roots, the heart was removed and embedded in OCT, frozen, and sectioned with a cryostat at 10 μm/section. The sections were stained with Oil red-O and counterstained with hematoxylin. The aorta was isolated from its origin in the heart to the ileal bifurcation. For *en face* lesion quantification, the aorta was cleaned by removing adventitial fat under a dissection microscope, then placed in 4% paraformaldehyde solution for 5 min, and washed in water. The fixed aorta was stained with a Sudan IV solution for 25 min, destained for 25 min in 70% ethanol, and washed in water. The aorta was then cut open longitudinally to expose the inner surface from the aortic arch through thoracic and abdominal aorta, embedded in glycerin, and sealed between a glass slides. Quantification of the area of the aortic plaques was performed with Image-Pro Plus version 4.1 software (Media Cybernetics, Inc., Bethesda, MD, USA).

### Flow Cytometry

Single cell suspensions from the aorta were prepared as described (6). Briefly, mice were anesthetized and perfused with PBS, and the aorta was removed from the aortic root to the branching of the iliac arteries. Aortas were cleaned from surrounding fat and cut in 1–2 mm pieces that were digested with 200 µg/ml Liberase DH (Roche) and 40 U/ml DNase I (NEB) for 25 min at 37°C. Spleen and lymph nodes (LN) (inguinal, axillary, and brachial) were removed from mice and gently meshed in PRMI1640 containing 10% FCS to prepare single cell suspensions. Anti-mouse antibodies used for FACS staining were Pacific Blue-coupled anti-CD4, PE-coupled anti-CD8a, PE/Dazzle™ 594 anti-CD8a, Pacific Blue-coupled anti-CD11b, PE/Dazzle™ 594 anti-CD11b, FITC-coupled anti-CD11c, Brilliant Violet 605-coupled anti-CD11c, FITC-coupled anti-CD19, APC-coupled anti-IL17A, Brilliant Violet 605-coupled anti-IL17A, Brilliant Violet 421-coupled anti-TCRβ, FITC-coupled anti-Ly6G PE-coupled anti-Ly6G, APC-coupled anti-B220, APC-coupled anti-F4/80, FITC-coupled anti-IFN-γ, APC-coupled anti-IFN-γ (all purchased from BioLegend), and APC-eFluor^®^ 780 anti-CD45, PE-coupled anti-γ/δ-T-cells (eBioscience). Live/Dead Fixable Aqua Dead Cell Stain was from Life Technologies. For intracellular cytokine staining, splenic, peripheral lymph node, and aortic single cell suspensions were cultured for 4 h with RPMI1640 containing 10% FBS, 100 IU/ml penicillin, 100 µg/ml streptomycin, 1 µM sodium pyruvate, 1× non-essential amino acids, 2.5 µM β-mercaptoehanol, and 2 μM l-glutamine, 50 ng/ml PMA, 750 ng/ml ionomycin (both Sigma-Aldrich) and GolgiStop (BD Bioscience). Intracellular staining for IFN-γ and IL-17A was performed using Fix&Perm^®^ cell permeabilization reagents (BD Biosciences). A BD FACSCanto™ II Flow Cytometry System (BD Bioscience) was used for acquisition of all the samples. FlowJo (Tree Star Inc., Ashland, OR, USA) was used to analyze the data.

### Immunofluorescence Staining of the Aortic Root

Frozen sections of aortic root were fixed with 4% PFA for 10 min, followed by permeabilization in 0.2% Triton X-100 for 8 min. The sections were blocked by 1% BSA and 1% donkey serum in PBS for 30 min. Sections were then stained with primary Abs: rabbit anti-citrullinated histone 3 (Abcam, ab 5103), rat anti-mouse Ly-6G (BioLegend), rat anti-mouse F4/80 (BioLegend), biotin conjugated hamster anti-mouse MCP-1 (BioLegend), goat anti-human/mouse MPO (R&D), hamster anti-mouse IL-1β (BioLegend), followed by staining with Alexa Fluor 488-conjugated donkey anti-mouse IgG, Alexa Fluor 488-conjugated donkey anti-goat IgG, Alexa Fluor 555-conjugated donkey anti-rabbit IgG and Alexa Fluor 568-conjugated donkey anti-hamster IgG, Alexa Fluor 647-conjugated donkey anti-rabbit IgG, or Alexa Fluor 594-conjugated donkey anti-rat IgG (all from Invitrogen) (Life Technologies). Cells and tissue sections were counterstained with DAPI (Life Technologies) before being mounted in ProLong Gold (Life Technologies) and examined by confocal microscopy.

### Quantification of Gene Expression in Aorta

The aorta branches were excised, cleaned, and homogenized with TRIzol reagent (Invitrogen) for RNA extraction using the Qiagen RNeasy kit. The cDNA was synthesized using BIO-RAD iScript reverse transcription supermix according to the manufacturer’s instructions. Quantitative real-time PCR was performed on BIO-RAD CFX96 Real-Time System thermocycler using specific TaqMan primers and probes (Life Technologies). *Gapdh* was used as the housekeeping gene to normalize the expression of the target genes, and the WT HFC-treated mice Ct was used in the delta delta Ct calculations for the determination of the fold gene expression. The primers (Assay ID) were as follows: Gapdh, Mm99999915_g1; IL-1β, Mm00434228_m1; Tnf-α, Mm00443258_m1; CCL2, Mm00441242_m1; CXCL1, Mm04207460_m1; CXLC2, Mm00436450_m1; IL-17A, Mm00439618_m1 (Thermo Fisher Scientific, Waltham, MA, USA).

### Quantification of Plasma Lipoproteins

Lipoproteins were quantified using commercial enzymatic methods (Wako Chemicals USA, Inc., Richmond, VA, USA).

### *In Vitro* Preparation and Analysis of NETs

To prepare NETs, 20 × 10^6^/ml BM isolated neutrophils were plated in 500 µl of serum-free RPMI containing 200 µM Ca^+^ in a 24-well tissue culture plate. Media alone (as control) or A23187 (10 µM) were added, and cells were incubated at 37°C for 5 h. The supernatant was removed and NETs were partially digested in RPMI containing 0.3 U/ml micrococcal nuclease for 10 min at 37°C, followed by centrifugation at 5,000 rpm for 5 min. Supernatants from media alone or supernatants from A23187 treatment were stored −20°C. To disrupt NETs, 40 µl DNase I (10,000 U/ml) (Sigma-Aldrich) was added to 500 µl supernatants from both conditions for 3 h at 37°C, as previously described. NETs were quantified by an ELISA detecting Cit-H3:DNA complexes, as previously described (22). Briefly, high-binding 96-well ELISA microplates were incubated overnight at 4°C with rabbit anti-citrullinated histone 3 (Abcam, ab 5103) in coating buffer from the Cell Death Detection ELISA kit (cat# 11544675001; Roche). After blocking with 1% BSA (cat# A7906; Sigma) in PBS, samples diluted with 1% BSA in blocking buffer were added, and plates were incubated overnight at 4°C, washed, and anti-DNA-POD (clone MCA-33; Roche) was added for 1 h at room temperature. Following incubation, TMB substrate (cat# T0440; Sigma) was added and absorbance was measured at 450 nm after addition of stop reagent (cat# S5814; Sigma).

### BM-Derived Macrophages (BMM) Preparation and Stimulation With NETs

Bone marrow cells were flushed from the femurs and tibiae of 8-week-old WT mice. These cells were cultured in RPMI 1640 medium containing 10% FBS and 50 ng/ml murine macrophage colony-stimulating factor for 7 days. For NETs stimulation, BMM were stimulated with UN-NETs, UN-NETs treated with DNase I, A23187-NETs, A23187-NETs treated with DNase I for 4 h. Gene expression levels of indicated cytokines/chemokines were determined.

### Statistical Analysis

Statistical analysis was performed using Mann–Whitney *U* test in GraphPad Prism (GraphPad Software, San Diego, CA, USA). Multiple comparisons were analyzed by one-way ANOVA. A *p* value less than 0.05 was considered significant.

## Results

### NETs Are Present in Atherosclerotic Lesions and Are Associated With the Development of Atherosclerosis

**Figure 1:**
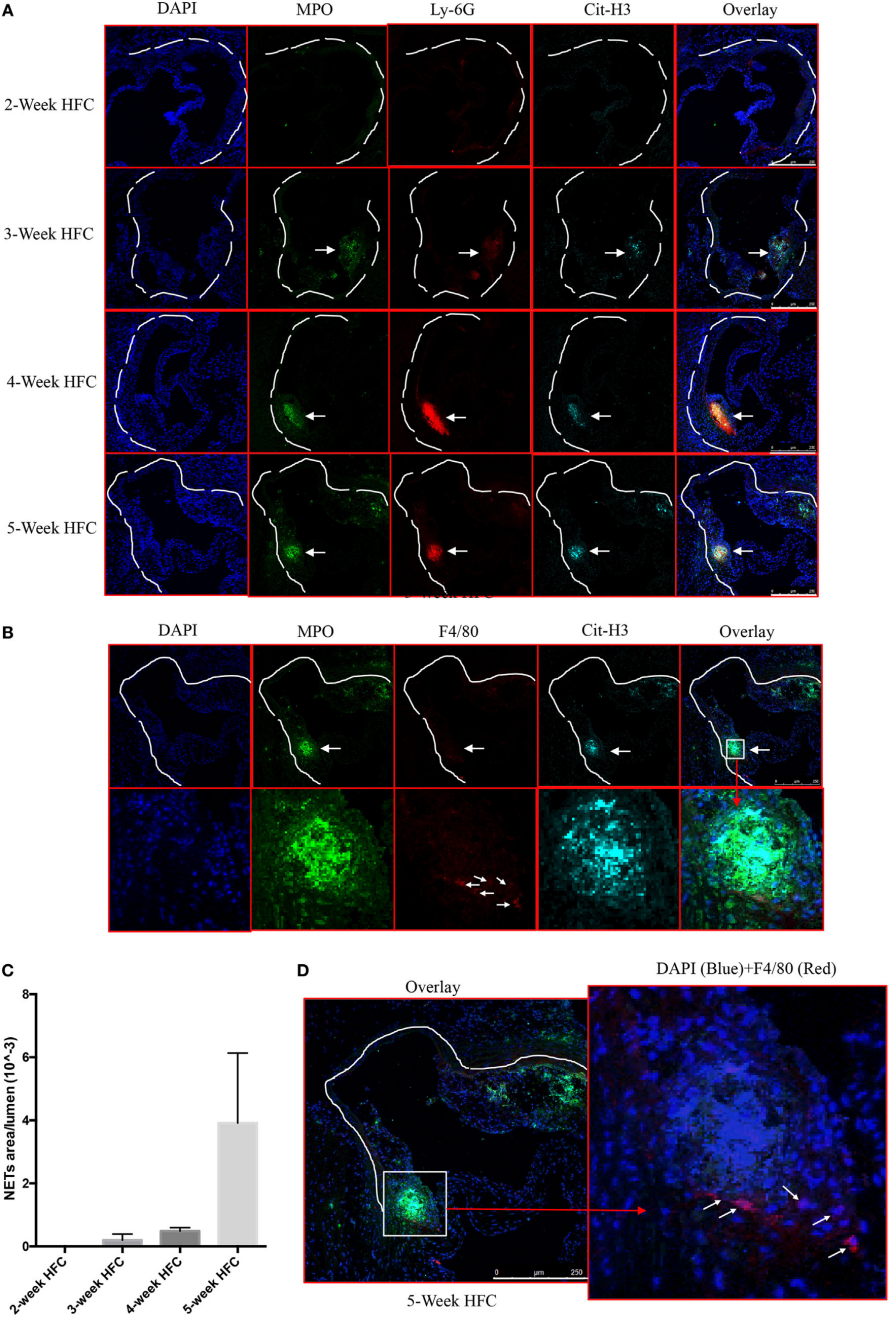
Neutrophil extracellular traps (NETs), but not macrophage extracellular traps (METs) are present in atherosclerotic lesions and are associated with the development of atherosclerosis. **(A)** WT mice were fed with high-fat chow (HFC) for indicated weeks, and aortic root sections were stained for NETs markers and observed by confocal immunofluorescence microscopy. Blue: DAPI, green: MPO, red: Ly-6G, and cyan: Cit-H3. Data are representative of four mice in each time point. NETs are indicated by white arrows. **(B)** WT mice were fed with HFC for 5 weeks, and aortic root sections were stained for METs markers (blue: DAPI, green: MPO, red: F4/80, and cyan: Cit-H3) and observed by confocal immunofluorescence microscopy. Lower panel represents higher magnification of the NETs area (white square) in the upper panel. Macrophages are indicated by white arrows. **(C)** Quantification of NETs formation from **(A)** (*n* = 4 per time points). Results represent the mean ± SEM. **(D)** Higher magnification of **(B)** for characterizing macrophages. Right panel represents overlay channels of DAPI (blue) and F4/80 (red) of the NETs area (white square) of left panel. Macrophages are indicated by white arrows.

We first examined whether NETs are present in atherosclerotic lesions in the WT mice following 2–5 weeks of HFC. Consistent with previous reports (2, 5, 6), analysis of the aortic root atherosclerotic lesions revealed extracellular fibrillar and web-like structures displaying co-localization of Ly-6G, citrullinated histone 3 (Cit-H3) and MPO consistent with NETs. These structures were readily detected as early as 3 weeks after HFC feeding, became more abundant at 4 weeks, and continued to be present in the plaque thereafter (Figures 1A,C). Recent studies suggest that macrophage extracellular traps (METs), which also contain Cit-H3, are present in several disorders (23) but we did not detect them in atherosclerotic lesions (Figure 1B). Macrophages were, however, detected in or close to NETs area (Figure 1D). These findings confirmed and extended the previous observations that NET structures are present in the atherosclerotic lesions of mice, even at early stages of atherosclerosis progression.

### Specific Deletion of PAD4 in the Myeloid Lineage Reduces Atherosclerosis Burden in Association With Diminished NET Formation

**Figure 2:**
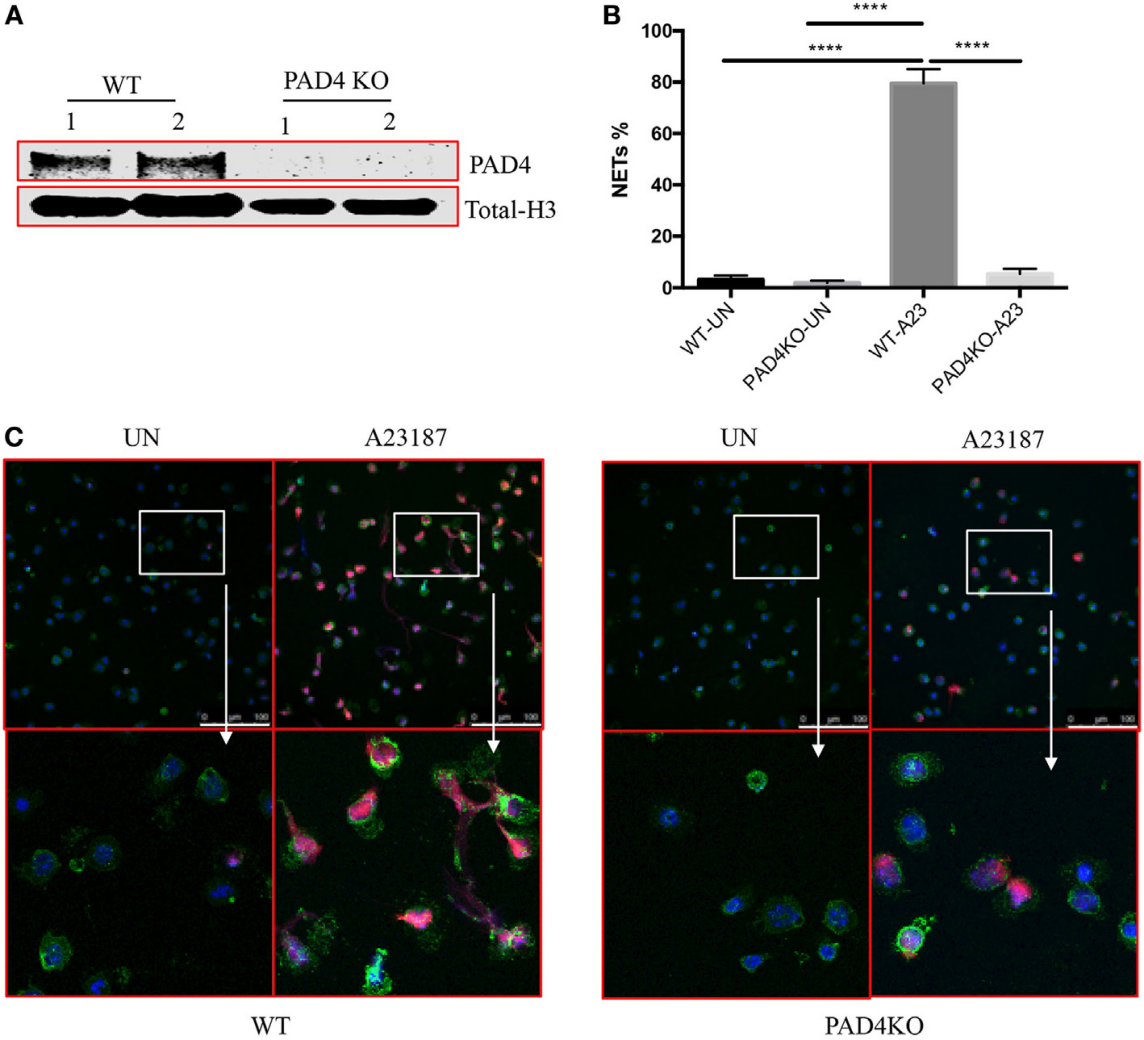
Myeloid peptidylarginine deiminase 4 (PAD4)-deficient neutrophils display impaired ability to generate neutrophil extracellular traps (NETs). **(A)** CD45^+^CD11b^+^Ly6G^+^ peritoneal neutrophils from WT and PAD4 KO mice sorted and lysed for western blot for PAD4. **(B)** CD45^+^CD11b^+^Ly6G^+^ peritoneal neutrophils from WT and PAD4 KO mice were stimulated with A23187 (10 µM) for 5 h or left unstimulated (UN). **(B)** Quantification of NETs formation (*n* = 3). **(C)** Representative immunofluorescence microscopy depicts NETs. DAPI: blue; green: neutrophil markers (CD45^+^CD11b^+^Ly6G^+^); red: Cit-H3. Bar: 100 µm. Lower panels are the 10× magnification of upper panels. Data are representative of three independent experiments. While neutrophils from WT mice display prominent NET formation with A23187, neutrophils from PAD4 KO mice do not form NETs ****p <0.001.

Given the significant presence of NETs containing Cit-H3 and the previous evidence that pan-PAD inhibitors mitigate plaque burden, we proceeded to investigate the specific role of myeloid PAD4 in the severity of atherosclerosis. We generated Apoe^−/−^LysMCre^+/−^PAD4^fl/fl^ mice (PAD4 KO), in which PAD4 is selectively deleted in cells of myeloid lineage, but not in other cells. First, we examined PAD4 protein from peritoneal neutrophils. Consistent with previous study (17), PAD4 protein was not detected in peritoneal neutrophils from PAD4 KO mice (Figure 2A). We next examined the effect of PAD4 deletion on NET formation. Peritoneal neutrophils from WT and PAD4 KO mice were isolated and stimulated *in vitro* with the ionophore A23187 to induce NETs. Consistent with previous findings in mice on the B6 background (9, 17), neutrophils from PAD4 KO mice were unable to generate these structures, whereas neutrophils from WT mice displayed evident NETs (Figures 2B,C).

**Figure 3:**
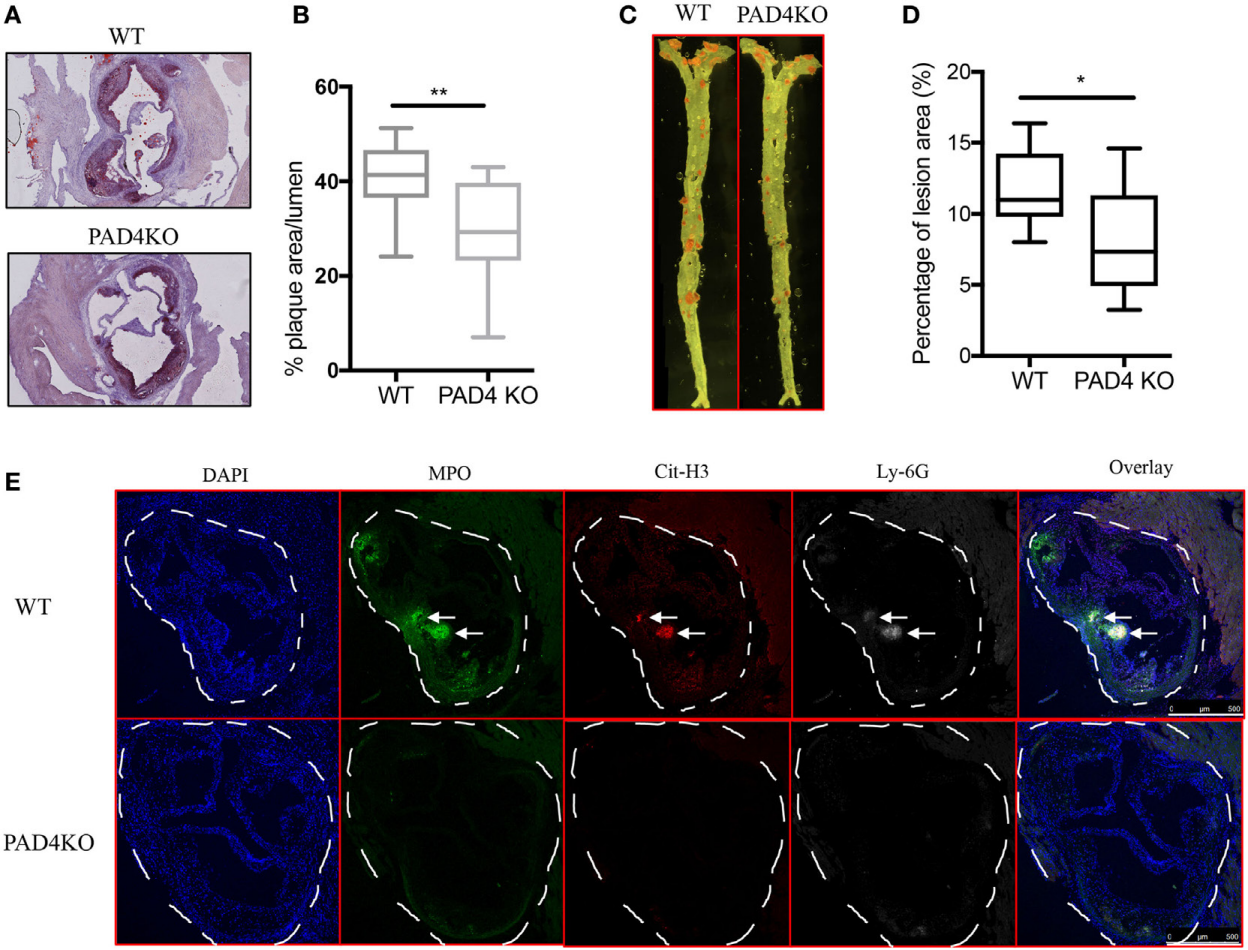
Lack of peptidylarginine deiminase 4 (PAD4) in myeloid lineage reduces atherosclerosis burden in association with diminished neutrophil extracellular trap (NET) formation in the artery. WT and PAD4 KO mice were fed high-fat chow (HFC) for 10 weeks. **(A)** Representative images of aortic root sections from WT and PAD4 KO mice stained with Oil Red O (red) and hematoxylin. *n* = 13–14/group in two independent experiments. **(B)** Quantitation of plaque area relative to the aortic lumen area of aortic root sections from WT and PAD4 KO mice placed on HFC for 10 weeks. **(C)** Representative images of *en face* preparations of intact aortas from WT and PAD4 KO mice placed on HFC for 10 weeks and stained with Sudan IV (Red) (*n* = 8–9/group in two independent experiments). **(D)** Quantitation of plaque area from the *en face* preparations of intact aortas from WT and PAD4 KO placed on HFC for 10 weeks. **(E)** Representative confocal immunofluorescence microscopy images of aortic root sections from WT and PAD4 KO mice placed on HFC for 10 weeks and stained for DNA (DAPI, blue), MPO (green), Cit-H3 (red), and Ly-6G (white). Areas of netting neutrophils are observed in the WT but not in the PAD4 KO mice. White arrows indicate NETs area. Data are representative of six mice in each group in two independent experiments. *p < 0.05, **p < 0.01.

After 10 weeks of HFC, WT mice and PAD4 KO mice displayed no significant differences in rates of weight gain (Figure S1A in Supplementary Material), ratios of spleen weight/body weight (Figure S1B in Supplementary Material) or levels of serum cholesterol and triglycerides (Figure S1C in Supplementary Material), indicating that PAD4 deficiency in myeloid cells did not affect the body weight and plasma lipid levels. By contrast, when compared to WT mice, PAD4 KO mice displayed a significantly lower arterial plaque burden, as shown by a significant reduction in lesion size in aortic root cross sections (Figures 3A,B) and significantly decreased plaque size in *en face* preparations of intact aortas (Figures 3C,D). The decrease in atherosclerosis burden observed in PAD4 KO mice was associated with significantly decreased NETs in atherosclerotic lesions (Figure 3E).

### Decreased Atherosclerotic Burden in PAD4 KO Mice Is Associated With Reduced Inflammatory Responses in the Aorta

**Figure 4:**
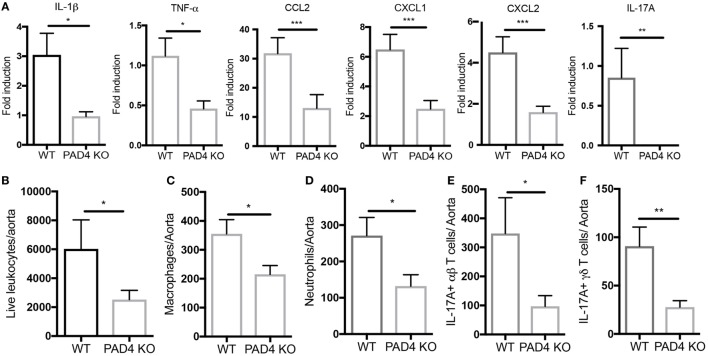
Lack of peptidylarginine deiminase 4 (PAD4) in myeloid lineage reduces proinflammatory responses in the aorta. **(A)** mRNA levels of IL-1β, TNF-α, CCL2, and IL-17A in the aortas from WT and PAD4 KO mice placed on high-fat chow (HFC) for 10 weeks. mRNA levels were normalized to *GAPDH* utilizing delta delta CT methods, and expressed relative to levels measured in one of the WT mice Results represent the mean ± SEM (*n* = 16–22/group from four independent experiments). Aortas from WT and PAD4 KO mice placed on HFC for 10 weeks were enzymatically digested, and total live aortic leukocytes **(B)**, macrophages **(C)**, neutrophils **(D)**, IL-17A-producing aortic αβT cells **(E)**, and IL-17A-producing aortic γδ T cells **(F)** were quantified by fluorescence-activated cell sorting. Results represent the mean ± SEM (*n* = 10–11/group from two independent experiments). *p < 0.05, **p < 0.01, ***p < 0.001.

**Figure 5:**
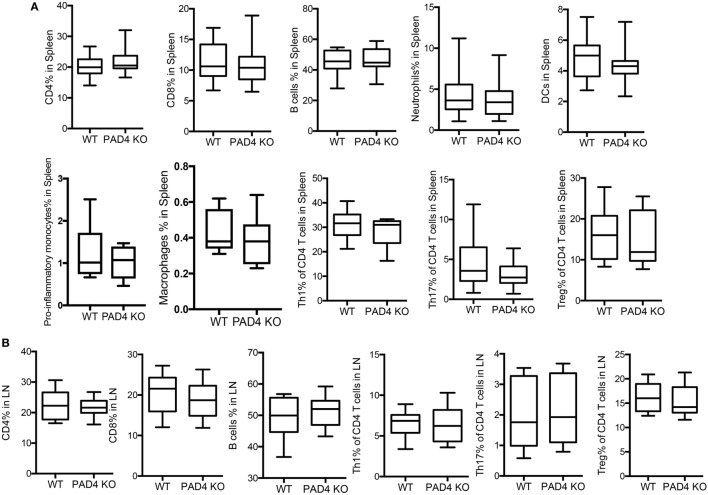
Lack of peptidylarginine deiminase 4 (PAD4) in myeloid lineage did not affect peripheral immune responses. WT and PAD4 KO mice were fed high-fat chow (HFC) for 10 weeks. **(A)** Percentages of CD4^+^ cells, CD8^+^ T cells, B cells, neutrophils, dendritic cells, proinflammatory monocytes (*n* = 6–7/group), macrophages (*n* = 6–7/group), Th1 cells, Th17 cells, and Tregs from spleens of WT and PAD4 KO mice (*n* = 13–14/group from two independent experiments) *p* = NS. **(B)** Percentages of CD4^+^ cells, CD8^+^ T cells, B cells, Th1 cells, Th17 cells, and Tregs from lymph nodes (LN) of WT and PAD4 KO mice (*n* = 9–10/group from two independent experiments), *p* = NS.

After 10 weeks of HFC, significantly decreased levels of proinflammatory mediators IL-1β, CCL2, CXCL1, and CXCL2 were detected in the aortas of PAD4 KO compared to WT mice (Figure 4A). This was confirmed by FACS analysis in aorta cell suspensions (Figure S2 in Supplementary Material), as significantly lower absolute numbers of total aortic inflammatory infiltrates were observed in PAD4 KO mice (Figure 4B). Previous studies have shown that CCL2, CXCL1, and CXCL2 contribute to the recruitment of neutrophils and macrophages to the lesions (24, 25). Indeed, absolute numbers of neutrophils and macrophages were significantly reduced in the aorta of PAD4 KO mice (Figures 4C,D). In addition, IL-17A mRNA levels (Figure 4A), absolute numbers of Th17 cells, and IL-17-producing γδ T cells (Figures 4E,F) were significantly decreased in PAD4 KO aortas compared to WT. Of note, alterations in immune cell populations between WT mice and PAD4 KO mice were only restricted to the aorta, as no significant differences in immune cell composition of the spleen and LN were observed between WT and PAD4 KO (Figure S3 in Supplementary Material; Figures 5A,B). These findings indicate that decreased atherosclerosis in PAD4 KO is associated with lower levels of proinflammatory cytokines and chemokines, and decreased arterial infiltration by macrophages, neutrophils and IL-17A-producing T cells.

### NETs Present in Atherosclerotic Lesions Stimulate Arterial Macrophages to Synthesize Inflammatory Mediators

**Figure 6:**
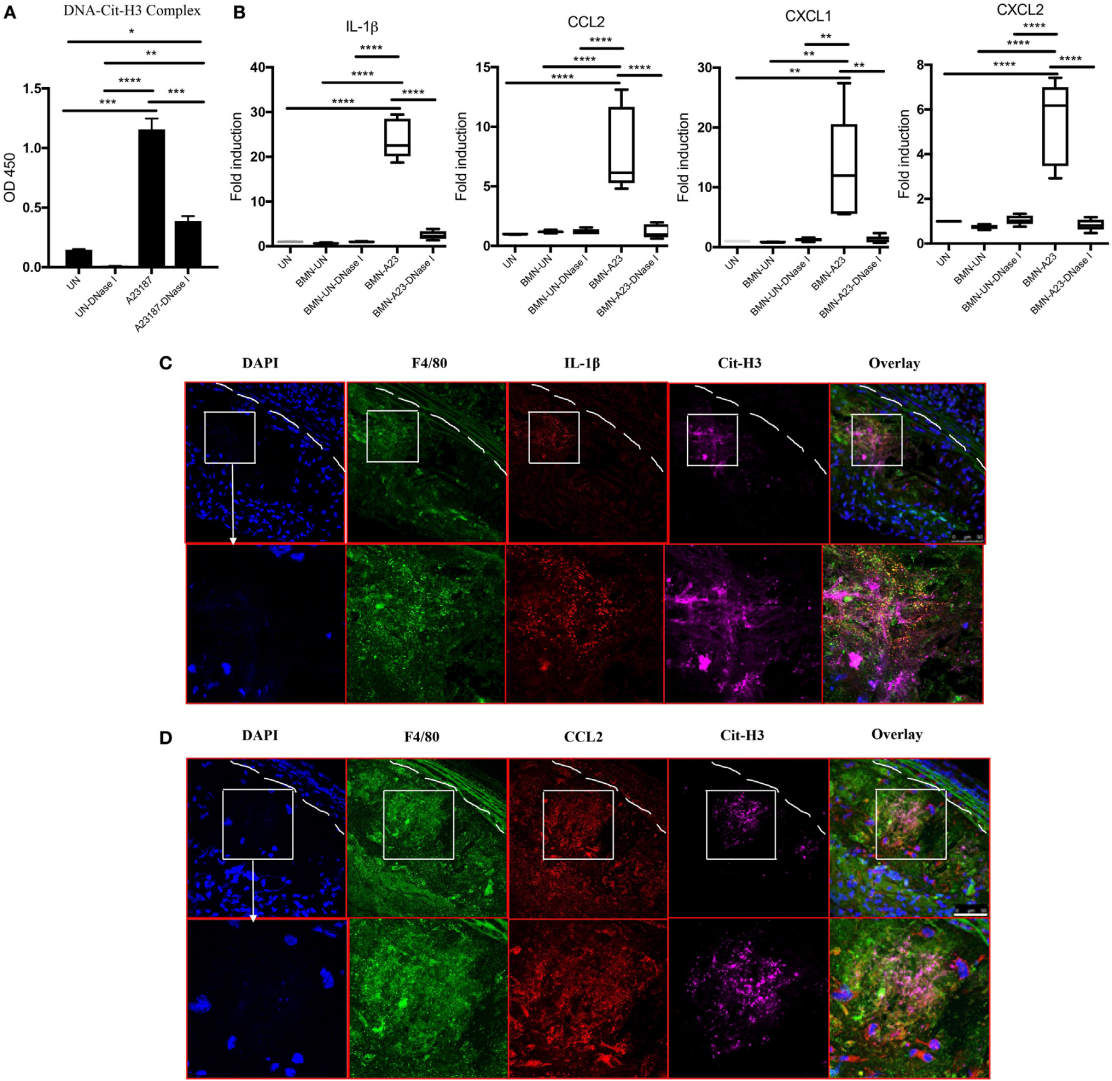
Neutrophil extracellular traps (NETs) present in atherosclerotic lesions stimulate inflammatory responses in arterial macrophages. **(A)** Bone marrow (BM)-derived neutrophils were stimulated in the absence (UN) or presence (A23187) of A23187 for 4 h. Half the UN-NETs or A23187-NETs were digested by deoxyribonuclease (DNase) I. NETs were quantified by measuring Cit-H3-DNA complexes on ELISA. **(B)** BM-derived macrophages were stimulated with UN-NETs (BMN-UN), UN-NETs treated with DNase I (BMN-UN-DNase I), A23187-NETs (BMN-A23), or A23187-NETs treated with DNase I (BMN-A23-DNase I) for 4 h. Gene expression levels of IL-1β, CCL2, CXCL1, and CXCL2 were determined. mRNA levels were normalized to *GAPDH* and expressed relative to levels measured in one of the BMN-UN conditions **(C)**. WT and peptidylarginine deiminase 4 (PAD4) KO mice were fed high-fat chow (HFC) for 10 weeks, and aortic root sections were stained for indicated markers and observed by confocal immunofluorescence microscopy. Lower panel represents enlarged area of the white squares in upper panels. Blue: DAPI, green: F4/80, red: IL-1β, and magenta: Cit-H3. Data are representative of four mice in two independent experiments. **(D)** WT and PAD4 KO mice were fed HFC for 10 weeks, and aortic root sections were stained for indicated markers and observed by confocal immunofluorescence microscopy. Lower panel represents enlarged area of the white squares in upper panels. Blue: DAPI, green: F4/80, red: CCL2, and magenta: Cit-H3. Data are representative of four mice in two independent experiments. *p < 0.05, **p < 0.01, ***p < 0.001, ****p < 0.0001.

Given that the presence of NETs in atherosclerotic lesions was associated with increased levels of various cytokines and chemokines and that NETs were found in close proximity to macrophages (Figure 1D), we determined whether NETs directly induce macrophages to synthesize these mediators. BM-derived neutrophils from WT mice were induced to form NETs by A23187 stimulation. Half of the generated NETs were exposed to DNase I as a negative control, as this enzyme disintegrates NETs. The formation of NETs and disruption of NETs by DNase I were quantified by ELISA measuring Cit-H3-DNA complexes (Figure 6A). *In vitro* exposure of syngeneic BM-derived macrophages to NETs led to significant induction of IL-1β, CCL2, CXCL1, and CXCL2, and this effect was abrogated by DNase I (Figure 6B). By contrast, there was minimal induction of these proinflammatory mediators by A23187 alone. The role of NETs in driving macrophage-driven inflammation was also supported *in vivo*, as IL-1β (Figure 6C) and CCL2 (Figure 6D) were detected in arterial macrophages in close proximity to NETs. Taken together, NETs can directly stimulate macrophages to synthesize inflammatory mediators important in arterial inflammation and plaque development.

### DNase I Treatment Decreases NETs and Ameliorates Atherosclerotic Burden in Apoe^−/−^ Mice

**Figure 7:**
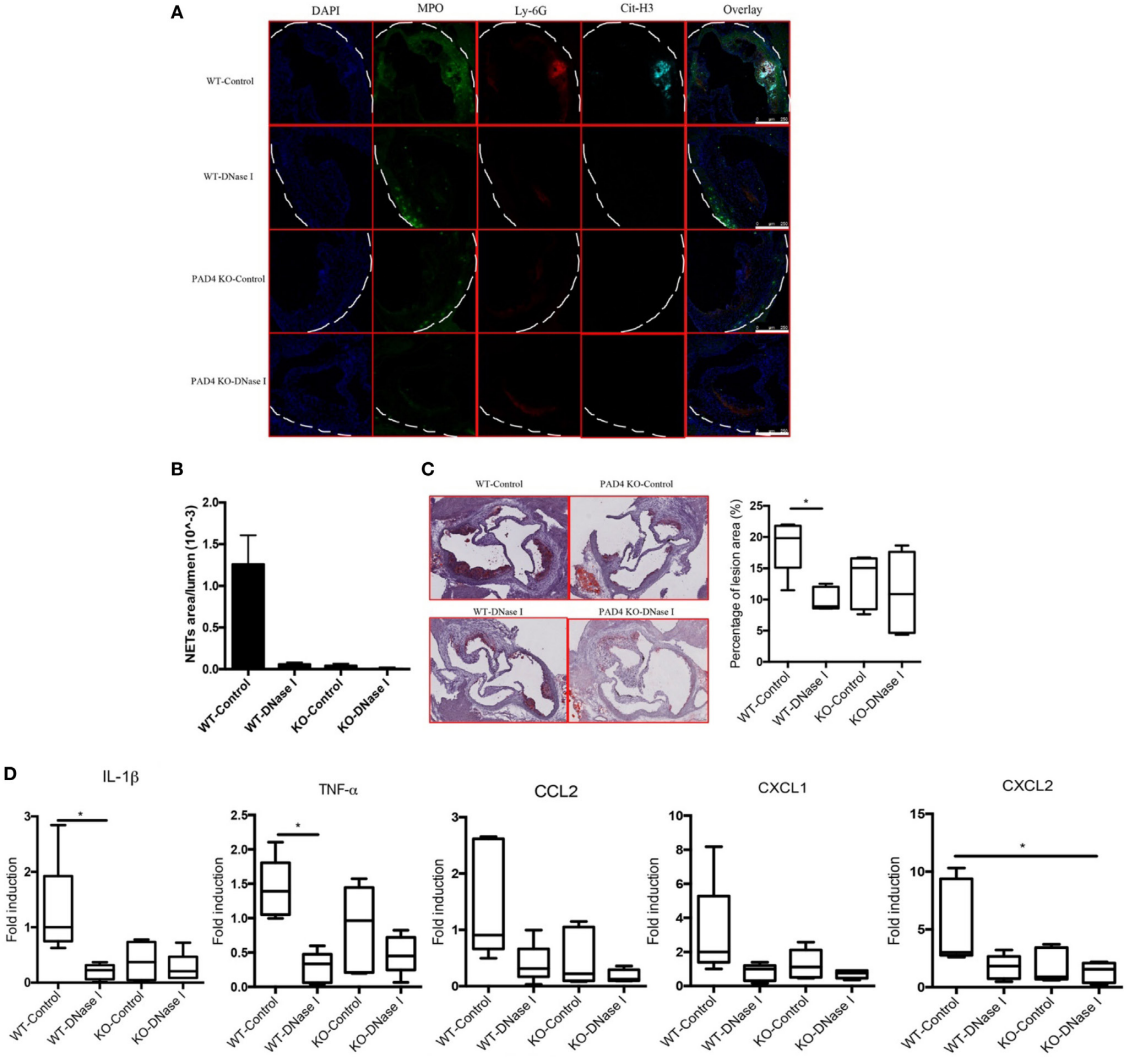
Deoxyribonuclease (DNase) I treatment abolished neutrophil extracellular traps (NETs) formation and ameliorated atherosclerotic burden. WT and peptidylarginine deiminase 4 (PAD4) KO mice were fed on high-fat chow (HFC) for 6 weeks, starting at 3-week HFC, 400 U of DNase I or vehicle control (PBS) was intravenously administered three times weekly until the end of experiments. **(A)** Representative confocal immunofluorescence microscopy images of aortic root sections stained for DAPI (blue), MPO (green), Ly-6G (red), and Cit-H3 (cyan). Data are representative of five mice in each group. **(B)** Quantification of NETs from **(A)** (*n* = 5/group). **(C)** Representative images of aortic root sections stained for lipid (Oil Red O, red) and hematoxylin (*n* = 5/group). **(D)** mRNA levels of IL-1β, TNF-α, CCL2, CXCL1, and CXCL2 in the aorta from WT and PAD4 KO mice placed on HFC for 6 weeks and administered with DNase I or vehicle control (PBS). mRNA levels were normalized to the GAPDH and expressed relative to levels measured in one of the vehicle control-treated WT mice (*n* = 5/group). *p < 0.05.

*In vivo* administration of DNase I, which can effectively degrade extracellular DNA present in NETs, has been used to dissolve NET structures and assess phenotypes driven by these lattices (23, 26–29). To further support the notion that NETs drive the development of atherosclerosis and that PAD4 KO mice have decreased atherosclerosis due to hampered NET formation, WT mice and PAD4 KO were administered DNase I. Treatment with DNase I did not modify rates of weight gain (or spleen weight/body weight ratios) (Figures S4A,B in Supplementary Material). By contrast, DNase I treatment decreased the number of NETs present in aortic root sections of WT mice and this associated with significantly reduced plaque size and decreased levels of IL-1β, TNFα, CCL2, CXCL1, and CXCL2 (Figure 7). By contrast, treatment with DNase I had minimal effect on NET formation or plaque size in PAD4 KO (Figure 7). Taken together, these results indicate that PAD4 expressed by myeloid cells modulates atherosclerosis development, at least in part, through the development of NETs.

## Discussion

Increasing evidence using different strategies (i.e., pharmacologically blocking NET formation, genetic deletion of NE and proteinase 3, etc.) suggests that NETs may play an important role in driving atherosclerosis. We now highlight the specific role of myeloid PAD4 and, more specifically, NET formation dependent on myeloid PAD4, in the pathogenesis of atherosclerosis. Our data also support the concept that NETs generated through PAD4-dependent mechanisms exert direct proinflammatory effects on macrophages leading to synthesis of cytokines and chemokines that can further amplify the inflammatory cascades in the artery.

Due to its nuclear localization, PAD4 mediates histone citrullination and has been found to contribute to chromatin decondensation during NET formation (8). Notably, citrullinated proteins are present in atherosclerotic plaques and co-localize with PAD4 within coronary artery plaques in humans, indicating that PAD4 may contribute to the citrullination process in atherosclerosis (30). In addition, we and others have also found that Cit-H3 is present in arterial plaques in animal models of atherosclerosis and Cit-H4 in human lesions (6, 31). Importantly, we found that Cit-H3 co-localized with MPO and Ly-6G, but not F4/80, in the aortas, thereby confirming that NETs, but not METs, are abundant in the atherosclerotic lesions and that their formation is dependent on PAD4.

Neutrophil extracellular traps could be readily detected in the atherosclerotic lesions as early as 3 weeks after HFC, while we observed minimal infiltrating arterial macrophages at the same time point. This finding suggests that NETs may play a critical role in the initial steps of the disease. Of note, recruitment of neutrophils has been shown to be especially important in plaque initiation (32). Indeed, arterial NET formation was evident by 3–4 weeks after starting HFC in this study. It is possible that, at earlier time points, aortic neutrophils are present as intact cells while during later stages a significant proportion of these cells extrude NETs to the extracellular space and amplify inflammation.

Multiple studies have shown that IL-1β plays important roles in atherosclerosis (33, 34). The pathogenic role of IL-1β is further confirmed by the recent Canakinumab Antiinflammatory Thrombosis Outcome Study, where targeting IL-1β led to a significantly lower rate of recurrent cardiovascular events (35). IL-1β was significantly reduced in myeloid-specific PAD4 KO mice, which is consistent with a previous report that NE^−/−^Proteinase-3^−/−^ mice, which displayed diminished NET formation, exhibited decreased aortic IL-1β (6). In that same study, human monocytes stimulated with NETs and subsequently treated with cholesterol crystals increased their synthesis of IL-1β (6), while cholesterol crystals alone did not affect IL-1β production (6). In our study using murine macrophages, NETs alone significantly increased IL-1β gene expression (6). In addition, we observed significantly decreased levels of IL-17 responses in PAD4 KO aortas, which is consistent with the notion that IL-1β may be critical in driving IL-17A responses (36, 37). We also found that NETs can directly stimulate macrophages to synthesize chemokines and this can amplify lesion formation by further recruiting myeloid cells. CCL2 and IL-8 were previously found to be highly expressed in human atherosclerotic lesions and postulated to be crucial in leukocyte recruitment into the arterial wall and developing lesions (38–40). It is interesting to note that IL-8 production by macrophages isolated from atherosclerotic plaques was significantly greater than IL-8 synthesis by autologous blood monocytes (41), supporting the hypothesis that NETs may locally stimulate macrophages to synthesize higher levels of chemokines.

To further confirm the pathogenic role of NETs in atherosclerosis, we administered DNase I to degrade formed NETs, and found that this intervention diminished NET formation and led to significant reductions in plaque size in WT mice when compared between vehicle-treated WT mice and DNase I treated WT mice. However, we did not observe significant differences between vehicle-treated PAD4 KO mice and DNase I-treated PAD4 KO mice. These findings are in agreement with a previous report showing that DNase I diminished NET formation in WT mice but not NE^−/−^PR3^−/−^ mice (6). Of note, coronary NET burden and DNase activity are predictors of ST-segment resolution and myocardial infarct size (MI) in humans (4). In murine models of myocardial ischemia/reperfusion (MI/R) injury, DNase I treatment led to a significant cardioprotective effects in Apoe^−/−^, but not in PAD4 KO mice (28). In addition, DNase I treatment also ameliorates cerebral I/R injury (42). Collectively, these data suggest that removal of NETs at the lesion site or preventing arterial NET formation may have therapeutic and/or preventive potential in atherosclerosis.

Interestingly, a recent study showed that PAD4 deficiency in hematopoietic cells did not modulate atherosclerotic plaque burden, which is different from our study (43). The discrepancies may be due to the differences in atherosclerotic murine model and the gender. In their study, the authors utilized Ldlr^−/−^ mice, while we employed Apoe^−/−^ background (43). In fact, the mechanisms by which the absence of ApoE and LDLr promote atherosclerosis are different (44). In addition, they utilized BM chimeric model by adoptive transfer BM from PAD4 KO mice (43), while we crossed PAD4 KO mice with Apoe^−/−^ mice, which may also add the confounding factor to this discrepancy (45, 46). Another important aspect related to the discrepancy is the gender differences. In the study by Franck et al., they only used male mice (43), while we only used females. Indeed, there is a known complex interaction between cytokine production and sex (44). Furthermore, in the study by Franck et al., they utilized 1.25% cholesterol (43), while we used 0.2% cholesterol. Thus, the differences in the HFC may also contribute to the discrepancy.

In summary, we propose that myeloid-specific PAD4 contributes to the development of atherosclerosis in a manner that is intimately linked to NET formation, with important effects on arterial innate and adaptive immune responses. Mechanistically, NETs locally stimulate arterial macrophages to synthesize proinflammatory mediators that promote IL-17A responses and facilitate further recruitment of myeloid cells. Taken together, our data suggest that NETs promote atherosclerosis and that the use of specific PAD4 inhibitors may have therapeutic benefits in this devastating condition.

## Ethics Statement

This study was carried out in accordance with the recommendations of ACUC. The protocol was approved by the “NIAMS ACUC.”

## Author Contributions

MK conceived the study. YL, CC-R, EM, NS, JK, MP, Z-HY, and AR conducted the experiments, prepared the figures, interpreted the data, and edited the manuscript. YL and MK wrote the manuscript. SH and KM provided the PAD4^fl/fl^ mice.

## Conflict of Interest Statement

The authors declare that the research was conducted in the absence of any commercial or financial relationships that could be construed as a potential conflict of interest.
