# Supplementary material for: Myeloid-Specific Deletion of Peptidylarginine Deiminase 4 Mitigates Atherosclerosis
Source: Front Immunol. 2018 Jul 26;9:1680. doi: 10.3389/fimmu.2018.01680 (PMC6094966; doi:10.3389/fimmu.2018.01680)
Supplement: Supplementary file 1 [file data_sheet_1.docx]

**
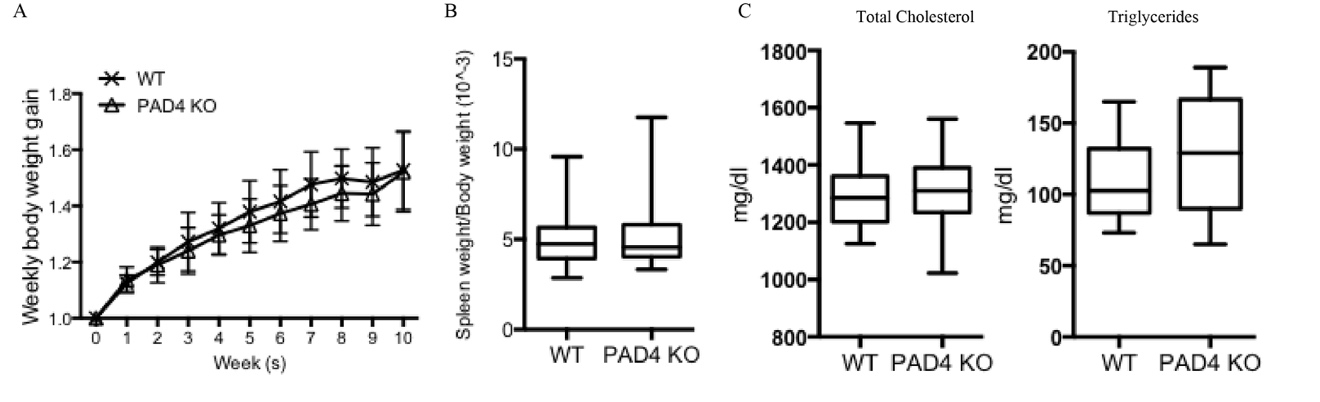
Supplemental Figure 1. Lack of myeloid PAD4 does not affect body weight or lipid levels.** Apoe-/-PAD4fl/fl and Apoe-/-LysMCrePAD4fl/fl mice were fed high-fat chow (HFC) for 10 weeks. **(A)** Weight gain (body wight of indicated week/initial body weight). Graphs represent mean + SEM of 20 animals per group. *p*=NS **(B)** Spleen weight/body weight (n=13-14/group) ; p=NS **(C)** Serum levels of total cholesterol and triglycerides (n=10/group); p=NS.

**
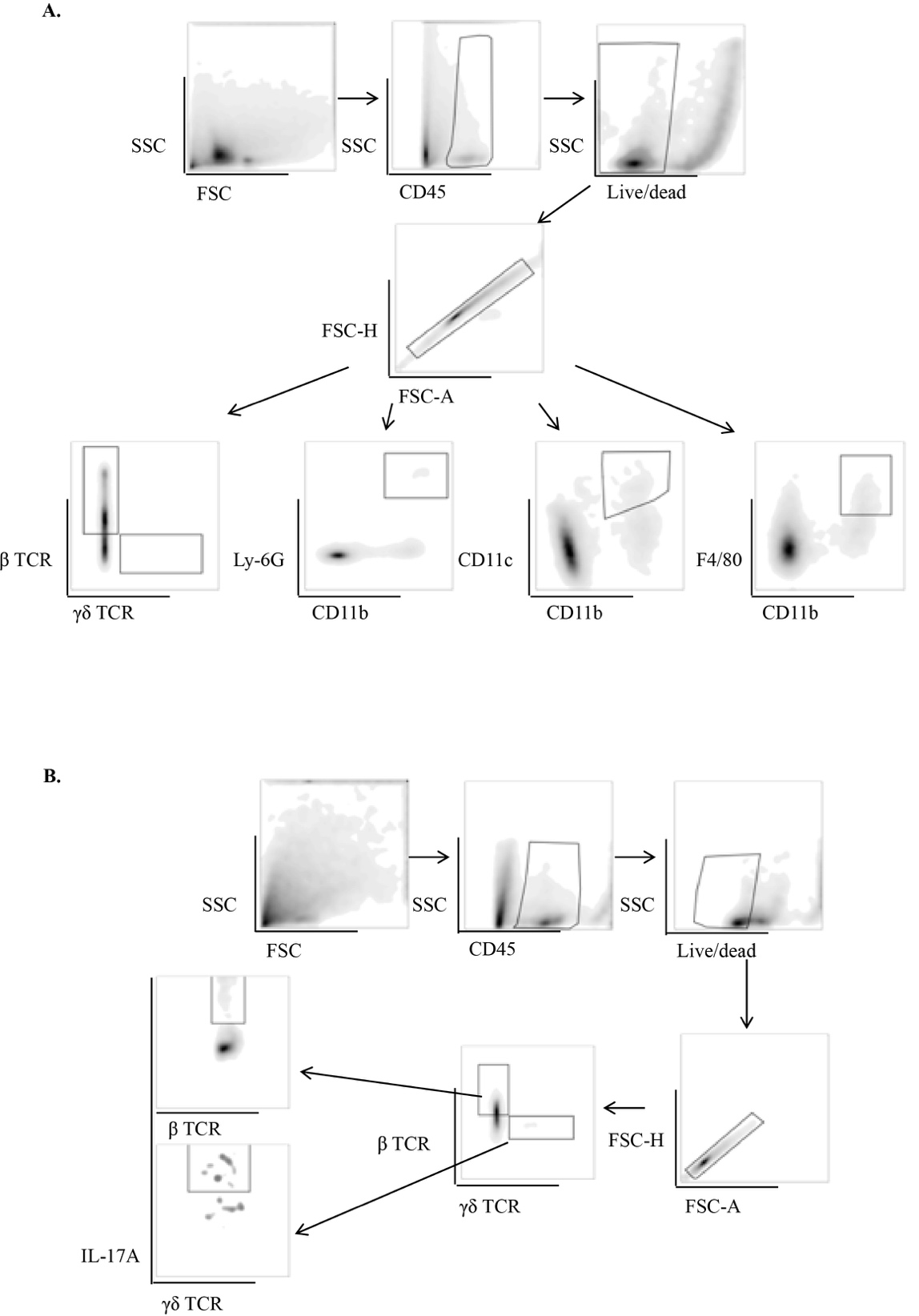
**

**Supplemental Figure 2. Gating strategy for Identification of aorta infiltrating immune cells. (A).** Enzymatically digested aortic cells were first gated on CD45+live cells, followed by gating on TCRβ T cells, TCR γδ T cells, CD11b+Ly-6G+ neutrophils, CD11b+CD11c+ DCs and CD11b+F4/80+ macrophages. **(B).** Enzymatically digested aortic cells were first gated on CD45+live cells, followed by gating on TCRβ T cells and TCR γδ T cells, and further gated on IL-17A-producing cells.

**
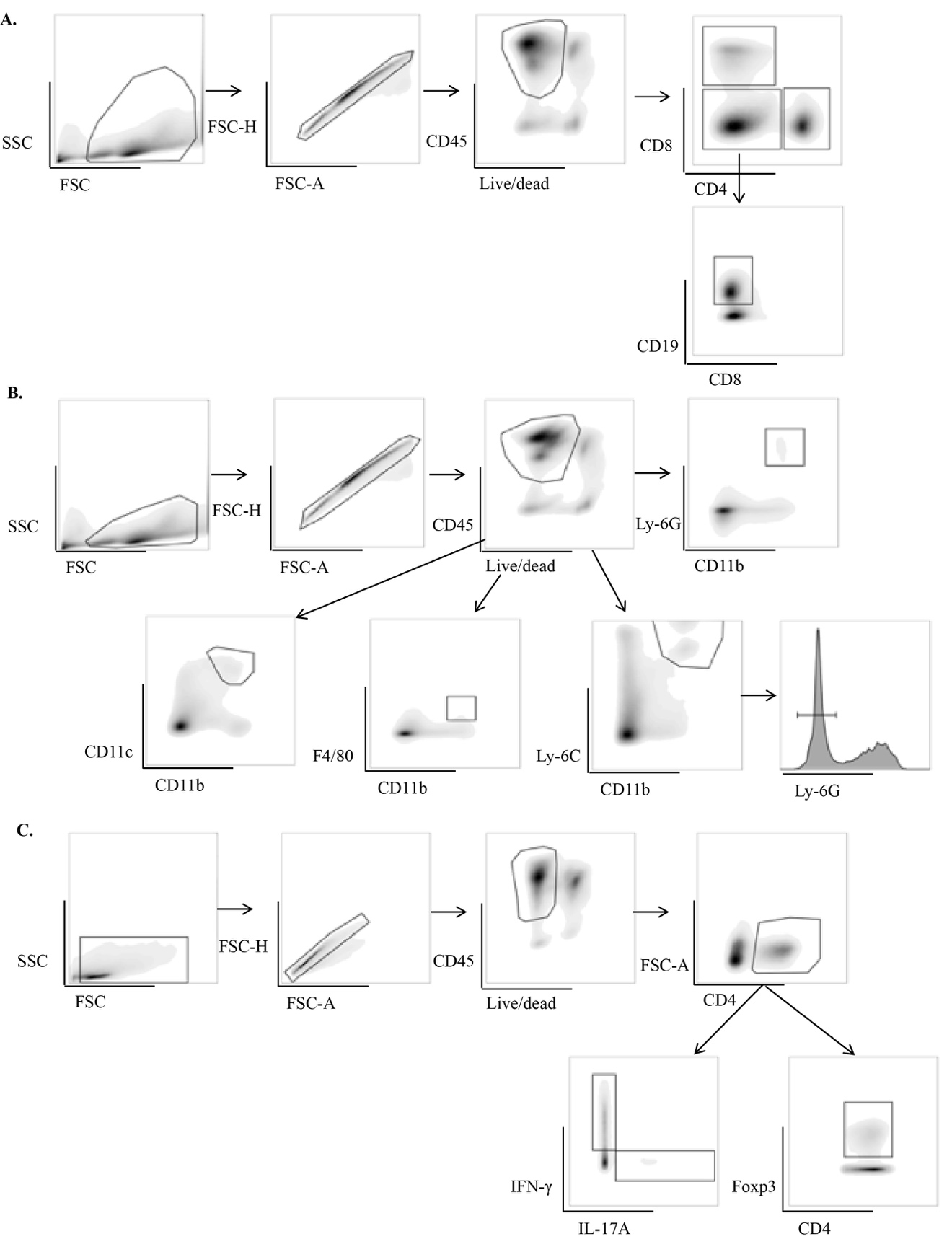
**

**Supplemental Figure 3. Gating strategy for Identification of immune subsets from spleen. (A).** Total splenocytes were first gated on CD45+live cells, followed by gating on CD4+ T cells and CD8+ T cells. CD19+ B cells were further gated from CD4-CD8- population. **(B).** Total splenocytes were first gated on CD45+live cells, followed by gating on CD11b+Ly-6G+ neutrophils, CD11b+CD11c+ DCs, CD11b+F4/80+ macrophages and CD11b+Ly-6C+Ly-6G- pro-inflammatory monocytes. **(C).** PMA and ionomycin stimulated splenocytes were first gated on CD45+live cells, followed by gating on CD4+ T cells, and then gating for IFN-γ+ CD4+T cells, IL-17A+CD4+T cells, and FoxP3+CD4+T cells.

**
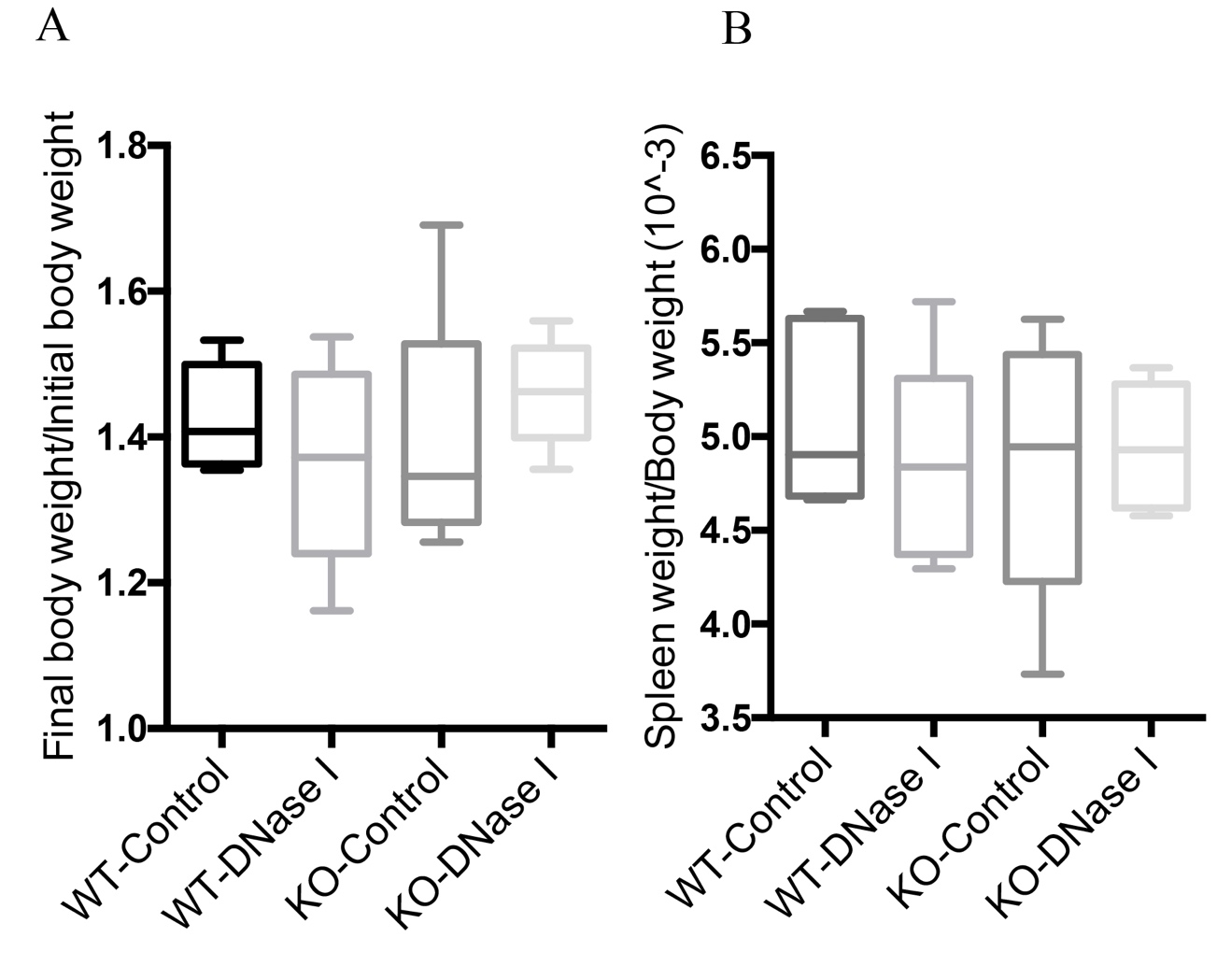
**

**Supplemental Figure 4. DNase I treatment did not affect the body weight and spleen weight.** Apoe-/-PAD4fl/fl and Apoe-/-LysMCrePAD4fl/fl mice were fed HFC for 6 weeks. Starting at week 3 of the HFC, 400 U of DNase I or vehicle control (PBS) were intravenously administered three times weekly until euthanasia. **(A)** Final body weight/initial body weight. **(B)** Spleen weight/body weight. n=5/group
